# Maekmoondong-tang in treatment of postoperative cough in patients with lung cancer: Study protocol for a randomized, double-blind, placebo-controlled, multicenter trial

**DOI:** 10.1097/MD.0000000000011541

**Published:** 2018-07-20

**Authors:** Chunhoo Cheon, Sohyeon Kang, Youme Ko, Mia Kim, Bo-Hyoung Jang, Yong-Cheol Shin, Seong-Gyu Ko

**Affiliations:** a Department of Preventive Medicine, College of Korean Medicine; b Department of Cardiovascular and Neurologic disease (Stroke Center), College of Korean Medicine, Kyung Hee University, Seoul, Republic of Korea.

**Keywords:** Bakumondo-to, cough, herbal medicine, lung cancer, Maekmoondong-tang, Mai-Men-Dong-Tang, study protocol

## Abstract

**Background:**

Cough is a common symptom that occurs in 25% of patients after lung cancer surgery. It might last a long time and degrade the quality of life of patients. Maekmoondong-tang (Bakumondo-to in Japanese or Mai-Men-Dong-Tang in Chinese) is a herbal medicine which has been widely used for respiratory diseases with cough in Korea, China, and Japan.

**Aims:**

The aim of the present study is to evaluate the efficacy and safety of Maekmoondong-tang for postoperative cough in patient with lung cancer.

**Methods/Design:**

This study is a randomized, double-blind, placebo-controlled, multicenter trial of Maekmoondong-tang. A total of 96 participants will be enrolled and allocated to 2 parallel groups: the Maekmoondong-tang group and the placebo group from 5 university hospitals. The participants will be administered either Maekmoondong-tang or a placebo 3 times a day for 4 weeks. The primary outcome measurement is the change in the Leicester Cough Questionnaire (LCQ) score. The secondary outcome measurements are the changes in the cough visual analog scale and Yin Deficiency Scale. The participants will visit 4 times in total for 4 weeks of trial period.

**Discussion:**

The present study will be the first multicener study to evaluate the efficacy and safety of Maekmoondong-tang for postoperative cough in patient with lung cancer surgery. The results of this study will provide a new treatment for cough using herbal medicine and will be a reference for planning clinical trial of herbal medicine in patient with cough.

## 1 Introduction

In 2015, cancer was responsible for 8.8 million deaths and of these, 1.69 million of them died from lung cancer.^[1]^ In Korea, lung cancer is the fourth most common cancer after thyroid cancer, gastric cancer, and colon cancer in 2014 and the 5-year cancer relative survival rates for the period 2010 to 2014 was 25.1%, an improvement of more than 5% over the period 2006 to 2010.^[2]^ It is reported that cough occurs in about 25% of patients after lung cancer surgery.^[3]^ Postoperative cough is known to be related to mediastinal lymph node resection, elevation of the diaphragm, loss of lung volume, and gastroesophageal reflux.^[4]^ Postoperative cough may last a long time and degrade the quality of life of patients, but there is not much research on this subject.^[5]^

Maekmoondong-tang (MMDT, Bakumondo-to in Japanese or Mai-Men-Dong-Tang in Chinese) is an herbal medicine approved by Ministry of Food and Drug Safety (MFDS). There are preclinical studies, clinical studies, and literature evidence that MMDT is effective in treating cough. Synopsis of Prescription of the Golden Chamber (Jin Gui Yao Lue) in which the MMDT was first introduced, records that MMDT treats cough with qi reflux and discomfort in the throat. Treasured Mirror of Eastern Medicine (Dongeuibogam) records that MMDT treats dyspnea due to fire which means serious cough caused by phlegm-fire or fire of thoroughfare vessel surging to lung. These records of classics of traditional medicine shows that MMDT is a herbal medicine that is effective against various respiratory diseases whose chief complaint is cough. In Korean medicine clinic, MMDT has been widely used for respiratory diseases with cough such as chronic bronchitis, bronchiectasis, and chronic laryngopharyngitis.^[6]^ Previous studies have shown that MMDT significantly relieves a cough and improves the quality of life in patient with cough after lung cancer surgery than the control group.^[7,8]^ It is also reported that MMDT alleviates cough in patients with chronic obstructive pulmonary disease (COPD).^[9]^ A systematic review of MMDT for cough reported that MMDT reduced the cough severity by 74% compared to the conventional cough medicines in various diseases.^[10]^ In addition to cough, the efficacy of MMDT in lung cancer itself, in which the tumor response rate was increased and the side effect rates were decreased in combination with EP regimen, has also been reported.^[11]^ There were several preclinical studies of MMDT on cough. MMDT attenuates airway hyperresponsiveness induced by ozone and has bronchodilating action by reducing the tension of bronchial smooth muscle and increasing cyclic AMP.^[12,13]^ MMDT also supressed the cough reflex caused by mechanical and chemical stimulations.^[14]^

In Korean medicine theory, cough is one of the symptoms due to yin deficiency which is a pathological change characterized by diminished moistening.^[15]^*Liriope Tuber* which is a primary constituent herb of MMDT has an effect of tonify yin and moisten the lung,^[16]^ therefore MMDT is expected to be effective for cough due to yin deficiency.

Many studies have been conducted for the improvement of quality of life and symptom management in patient with cancer lately. However, study for cough after lung cancer surgery is rarely conducted relative to its frequency.

The aim of the present study is to evaluate the efficacy and safety of MMDT for cough after lung cancer surgery. For this, a randomized, double-blind, placebo-controlled, multicenter trial will be conducted.

## 2 Materials and methods

### 2.1 Study design

**Figure 1:**
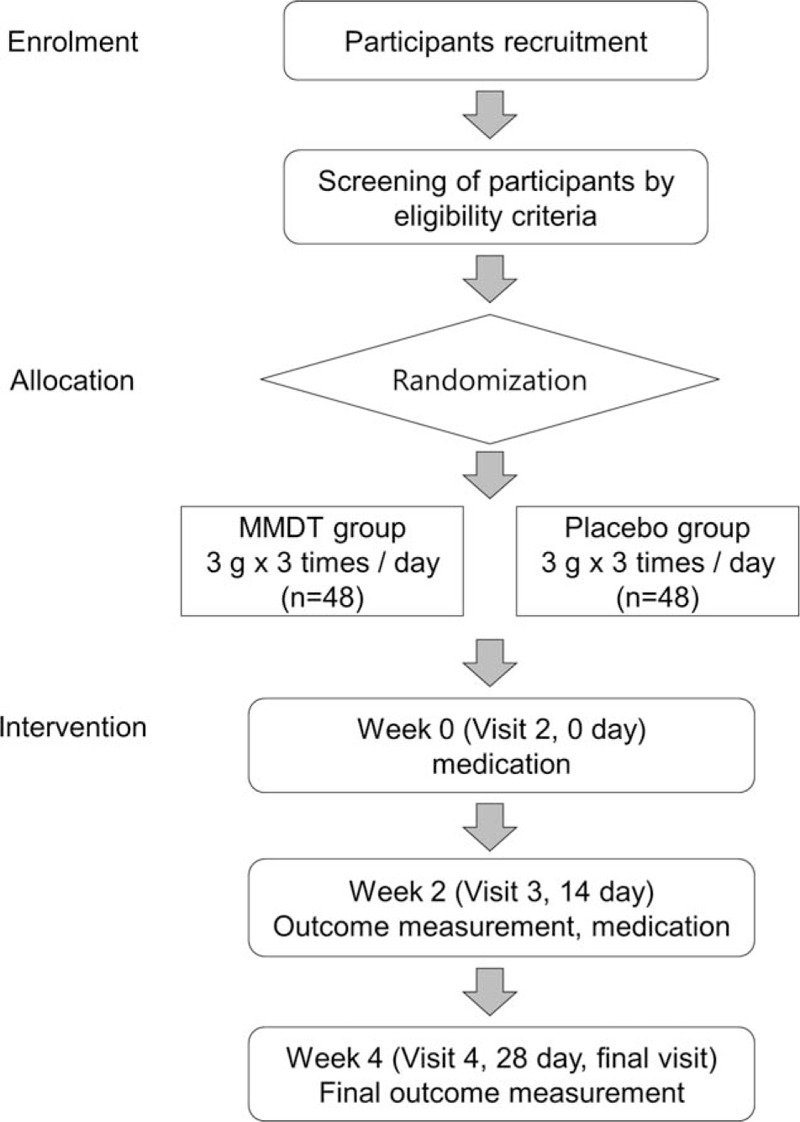
Study flowchart.

A randomized, double-blind, placebo-controlled, multicenter trial will be conducted at the Korea University Guro Hospital, Korea University Anam Hospital, Korea University Ansan Hospital, Gangnam Severance Hospital, and Inje University Ilsan Paik Hospital. Participants who meet the eligibility criteria will be enrolled and randomly allocated to MMDT group or placebo group. Each participants will visit the trial institutions 4 times for 4 weeks. At each visit, participants will be examined for signs and symptoms related to cough, quality of life and safety. The study flow chart is shown in Figure 1. Protocol modifications are not expected; however if they are necessary, any changes in the protocol will be announced to the all investigators via a conference. The management, analysis, and reporting of study will be conducted independently by the study investigators.

### 2.2 Participants

The inclusion criteria are as follows:

- 20 years or older;
- patients undergone segmentectomy or lobectomy for lung cancer within 1 month;
- patients who do not or poorly respond to one week administration of common antitussive agents;
- Eastern Cooperative Oncology Group (ECOG) performance status 0 to 2;
- participants who are willing and able to give informed consent for participation in the study.

The exclusion criteria are as follows:

- patients undergoing adjuvant chemotherapy;
- patients who have been diagnosed with acute respiratory disease within 1 month;
- patients who have been diagnosed with bronchial asthma or bronchiectasis within 1 year;
- patients taking angiotensin converting enzyme inhibitor;
- patients diagnosed pneumonia with a positive chest x-ray;
- patients with infection suspected to the cause of cough in sputum cultures;
- patients with pseudoaldosteronism;
- participants who have known prior hypersensitivity to any investigational product component;
- patient with acute or chronic infections requiring treatment (active hepatitis A, B, and C viruses, human immunodeficiency virus, tuberculosis);
- pregnant or lactating females;
- women of childbearing potential;
- patients who do not agrees to use effective means of contraception and not to donate sperm during the trial and up to 1 month after final administration;
- patient who participated other clinical trials of medicine or medical devices within 1 month; and
- individuals who are judged inappropriate for the study by investigator

The withdrawal criteria are as follows, and the reasons of the withdrawal will be clarified in the case report forms (CRFs):

- participants who do not follow the instructions of investigators;
- occurrence of significant protocol violations;
- occurrence of a serious adverse event (AE);
- investigator's decision to terminate the process for the sake of the participant's health; and
- participant's withdrawal of consent

The participants who are withdrawn from the study after allocation will be followed for outcome measurements as far as possible.

### 2.3 Recruitment

Participants will be recruited through 2 routes. Patients who visit the trial institutions and fulfil the eligibility criteria will be recommended by the investigators. Patients who see the trial poster on bulletin boards of the institutions will contact the investigators voluntarily. Written informed consent will be obtained from all participants prior to enrolment by the investigators.

### 2.4 Sample size

Sample size calculation was conducted based on previous study that reported efficacy of MMDT on prolonged cough after lung cancer surgery.^[7]^ There was no study evaluated efficacy of MMDT using Leicester Cough Questionnaire (LCQ) in patients with lung cancer, thus, the changes of Short-From 36 (SF-36) score of the previous study were transformed to LCQ score. The mean differences of LCQ scores were hypothesized to be 8.40 and 5.52 in MMDT group and placebo group, respectively, and the standard deviation was hypothesized to be 3.45. The following formula was used to calculate the sample size. The calculation was performed using 95% power, and a 5% significance level 
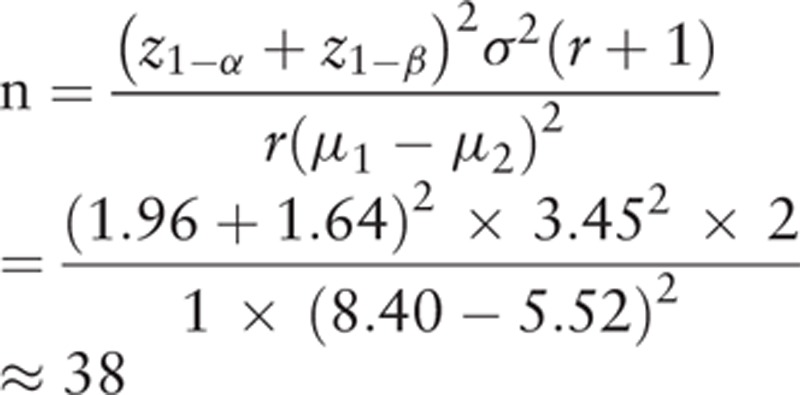


Considering a drop-out rate of 20%, the needed sample size is approximately 48 participants for each group. Therefore, a total of 96 participants are needed for the present study.

### 2.5 Randomization and allocation

The enrolled participants will be randomized using random numbers generated by computer at an independent centre, Institute of Safety, Efficacy and Effectiveness Evaluation for Korean Medicine (ISEE). The study participants will be assigned to one of 2 groups with a 1:1 allocation ratio in blocks of 4 by investigators in each trial institutions. The randomization table will be kept in an opaque sealed envelope and maintained by the ISEE during the trial period. The randomization table should be opened according to Standard Operating Procedures (SOPs).

### 2.6 Blinding

A researcher not involved in recruitment, intervention or outcome assessment will handle the randomization. Therefore, both the investigators and the participants will be blinded for assignment of the investigational drugs. The investigational products will be manufactured and labeled at the Hanpoong Pharm & Foods Co, Ltd, a pharmaceutical company.

### 2.7 Treatment protocol

The participants will receive MMDT or a placebo drug for 4 weeks. They will take 3 grams of granules by mouth with water 3 times a day before or between the meals for 4 weeks. The daily dose and usage follow the recommendation of the MFDS on MMDT. The participants will be required to return any remains of drug for calculating compliance. The participants will be prohibited to receive other additional treatment for cough during the trial.

### 2.8 Interventions

Hanpoong Pharm and Foods Co., Ltd. produces the MMDT and placebo according to Korea Good Manufacturing Practice (KGMP) standards. The MMDT is a light brown-colored granuels, and it consist of 3.33 g *Liriope Tuber*, 1.67 g *Pinellia Tuber*, 3.33 g *Oryzae Semen*, 1 g *Zizhyphi Fructus*, 0.67 g *Ginseng Radix Alba*, 0.67 g *Glycyrrhizae Radix et Rhizoma*. The placebo is made of corn starch, lactose hydrate, citric acid hydrate, caramel colorant and ginseng flavor powder, and it is similar in appearance, shape, weight, taste, and color to the MMDT.

### 2.9 Primary outcome measurement

The primary outcome in the present study is the change in the LCQ score between the baseline (Visit 2) and after the treatment (Visit 4). The LCQ is a valid, repeatable cough-specific quality of life questionnaire consists of 19 items, each rated on a 7-point Likert scale.^[17]^ Korean version of the LCQ also has been validated its concurrent validation, internal consistency, repeatability, and responsiveness.^[18]^ The total score of LCQ is 3 to 21, and a higher score means a better quality of life. The LCQ will be measured at visit 2, 3, and 4.

### 2.10 Secondary outcome measurement

Secondary outcome measurements include the changes in the cough visual analog scale (VAS) and Yin Deficiency Scale (YDS). The study participants will mark a 100-mm linear VAS with 0 representing “no cough’ and 100 representing “worst cough.” YDS is a validated questionnaire measuring yin deficiency, consists of 27 items, each rated on a 7-point Likert scale.^[15]^ The cough VAS and YDS will be measured at visit 2, 3, and 4. The study schedule is detailed in Table 1.

**Table 1:**
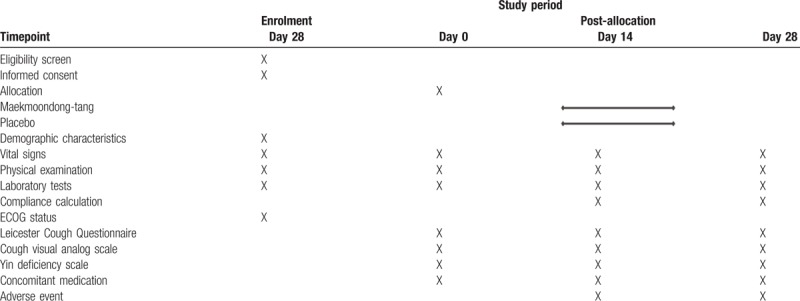
Study schedule of the Maekmoondong-tang study.

### 2.11 Safety outcomes

All variables related to the participants’ safety including vital signs, physical examination, hematologic test, biochemical test, and urine test will be documented on the CRFs at every visit. Any AEs that occur after taking investigational drug will be documented on the CRFs. If the AE is severe and related to the investigational product, the participant will be withdrawn from the study, and the appropriate treatment and reimbursement will be provided to them.

### 2.12 Statistical analysis

#### 2.12.1 Efficacy assessment

The baseline characteristics will be analyzed by a *t* test for continuous variables or a chi-square test (Fisher's exact test will be conducted when the expected value is < 5) for categorical variables. If the normality assumption is not satisfied for the continuous variables, Mann-Whitney *U* test will be used. The last observation carried forward (LOCF) method will be used for missing values. For the efficacy assessment, an independent *t*-test will be conducted to compare the mean differences between the baseline and visit 4 for the 2 groups. In addition, paired *t*-test will be conducted to see intergroup changes between baseline and visit 4. Statistical analyses for efficacy will be conducted for both the ITT (intention-to treat, all randomized participants who administered at least single dose of investigational product), and PP (per-protocol, participants who completed the study without any major protocol deviations). The ITT analyses will be the primary analyses and PP analysis will be for reference. If the *P* value is <.05, the result will be considered as statistically significant. There is no plan for interim analysis.

#### 2.12.2 Safety assessment

All the safety related variables such as vital signs and laboratory test results will be compared between MMDT group and placebo group at every visit. We will check whether there is a significant difference between the 2 groups by *t*-test and whether there is a change beyond the normal range. The incidence of AEs will be compared by chi-square test. The statistical analyses for safety will be conducted by ITT analyses.

### 2.13 Data and safety monitoring

Pharm data, the contract research organization (CRO), will monitor this trial. Each institution participating in the present study will be monitored while this study is in progress. Auditing is not scheduled for this study. Double data entry and range checks will be conducted for improvement of data quality. AEs will be reported to IRBs, and suspected unexpected serious adverse reaction will be reported to MFDS.

### 2.14 Ethics and dissemination

The present study has been approved by the Institutional Review Boards (IRBs) of all 5 institutions: the Korea University Guro Hospital(KUGH17284-001), Korea University Anam Hospital(ED17065), Korea University Ansan Hospital (AS17184-002), Gangnam Severance Hospital (3-2018-0058), and Inje University Ilsan Paik Hospital (ISPAIK 2017-11-026-001). The current protocol version is 1.1. Any information acquired from study participants will be dealt with confidentially. During the entire study, data will be handled by the participant identification code which is assigned to each participant at enrolment. All records from the study will be stored securely in locked cabinets or in password-protected electronic documents. Only investigators in charge will have the right to access the data. The results of the present study will be published in a scientific journal or presented through a scientific conference. There is no plan to public release the full protocol and personal datasets.

## 3 Discussion

Cough is a symptom of many respiratory diseases such as tonsillitis, laryngitis, pharyngitis, lung cancer, asthma, bronchitis, pneumonia, and tuberculosis. A systematic review reported that several herbal medicines have positive effect on cough in patient with childhood cough variant asthma.^[19]^ However, that study only evaluated the efficacy of combination treatment of herbal medicines and conventional therapies, and excluded the patients with serious respiratory disease including chronic obstructive pulmonary disease and lung cancer. There are several clinical trials to investigate efficacy of herbal medicine for cough in patient with cough variant asthma, postinfectious cough, and allergic asthma.^[9,20,21]^ Although previous study showed that MMDT can improve chronic cough after lung cancer surgery, that study had a limitations of small and single-centre trial.^[7]^ A study protocol for the treatment of chronic dry cough using MMDT has already been published^[22]^; however, the present study has several distinctive features of relatively large scale, multicenter trial for patients with lung cancer. The present study is the first multicenter, randomized, controlled trial for evaluating the efficacy of MMDT in Korea. The results of this study might provide a new treatment for postoperative cough in patient with lung cancer surgery. We expect that the present study protocol will serve as a reference for planning further clinical trials to treat cough using herbal medicines.

## Acknowledgments

We wish to acknowledge Hanpoong Phar. & Foods Co. Ltd. for providing investigational product support.

## Author contributions

**Funding acquisition:** Seong-Gyu Ko.

**Methodology:** Youme Ko, Mia Kim.

**Project administration:** Seong-Gyu Ko.

**Supervision:** Seong-Gyu Ko.

**Writing – original draft:** Chunhoo Cheon, Sohyeon Kang.

**Writing – review & editing:** Bo-Hyoung Jang, Yong-Cheol Shin.
